# Robust Mesoporous SiO_2_-Coated TiO_2_ Colloidal Nanocrystal with Enhanced Adsorption, Stability, and Adhesion for Photocatalytic Antibacterial and Benzene Removal

**DOI:** 10.3390/ma18163844

**Published:** 2025-08-15

**Authors:** Nan Xiao, Aijia Zhang, Kunjie Yuan, Wenbin Cao

**Affiliations:** 1 School of Materials Science and Engineering, University of Science and Technology Beijing, Beijing 100083, China; 13982936009@163.com (N.X.); 19134020402@163.com (A.Z.); 2 School of Energy and Environmental Engineering, Hebei University of Technology, Tianjin 300401, China; 3 Tianjin College, University of Science and Technology Beijing, Tianjin 301830, China

**Keywords:** SiO_2_-coated TiO_2_, photocatalysis, oxygen vacancies, benzene removal

## Abstract

The utility of nanostructured TiO_2_ in the degradation of organic compounds and the disinfection of pathogenic microorganisms represents an important endeavor in photocatalysis. However, the low photocatalytic efficiency of TiO_2_ remains challenging. Herein, we report a robust photocatalytic route to benzene removal rendered by enhancing its adsorption capacity via rationally designed mesoporous SiO_2_-coated TiO_2_ colloids. Specifically, amorphous, mesoporous SiO_2_-coated TiO_2_ nanoparticles (denoted T@S NPs) are produced via a precipitation-gel-hydrothermal approach, possessing an increased specific surface area over pristine TiO_2_ NPs for improved adsorption of benzene. Notably, under UV irradiation, the degradation rate of benzene by T@S NPs reaches 89% within 30 min, representing a 3.1-fold increase over that achieved by pristine TiO_2_. Moreover, a 99.5% degradation rate within 60 min is achieved and maintains a stable photocatalytic activity over five cycles. Surface coating of TiO_2_ with amorphous SiO_2_ imparts the T@S composite NPs nearly neutral characteristic due to the formation of Ti-O-Si bonds, while manifesting enhanced light harvesting, excellent stability, adhesion, and photocatalytic bacteriostatic effects. Our study underscores the potential of T@S composites for practical applications in photocatalysis over pristine counterparts.

## 1. Introduction

Among various photocatalytic materials (oxides, carbides, etc.), TiO_2_ has been widely used to decompose volatile organic compounds (VOCs), disinfect bacteria and viruses, etc. owing to its hydrophilicity, antifouling, photodegradability, and antibacterial property [1,2]. However, the limitations of TiO_2_, including its large band gap and low quantum efficiency, hinder its industrial viability. To date, many efforts have been devoted to modifying TiO_2_, including transition metal ion doping [3,4], semiconductor compounding [5,6], precious metal precipitation [7], organic-dye sensitization [8], and non-metal ion doping [9], thereby improving light harvesting and photocatalytic performance. Notably, the adsorption technology represents an important approach to VOC degradation, where mass transfer limits the photodegradation rate. Thus, the ability to enhance the adsorption of organic pollutant molecules enables their improved contact with photocatalysts [10].

TiO_2_-based composites in either film [11] or powder form [12,13,14,15] as photocatalysts have been found to effectively decompose organic compounds in liquid and gas phases. On the other hand, the ability to create TiO_2_-based composites in a colloidal form offers new opportunities for use in concrete [16] and glass [17] as building nanomaterials. Notably, various synthetic routes to TiO_2_/SiO_2_ nanocomposites have been reported, including grinding and calcination [18], co-precipitation and calcination [19], and a sol-gel approach [14,15,18]. However, most of the abovementioned methods require a multistep process and high-temperature treatment to obtain a highly crystalline TiO_2_ [20].

Herein, we report the crafting of mesoporous SiO_2_-coated TiO_2_ colloids to markedly improve the adsorption of non-polar vapor phase pollutant benzene and photocatalytic performance. Specifically, we developed a simplified precipitation-peptization-hydrothermal approach to synthesize core-shell structured SiO_2_-coated TiO_2_ nanoparticles (20–30 nm) at 120 °C, which demonstrated effective adsorption capability toward non-polar gaseous benzene pollutants. Although such composite materials have been extensively studied, the underlying mechanism for their enhanced performance remains insufficiently explored. This study systematically investigates: (1) The resulting T@S NPs display enhanced adsorption of benzene over that of pristine TiO_2_ NPs. A suite of characterizations, including transmission electron microscope (TEM), X-ray photoelectron spectroscopy (XPS), Fourier transform infrared spectroscopy (FTIR), and electron paramagnetic resonance (EPR), was performed to scrutinize the mechanism of boosted photocatalytic performance of T@S composites for low-concentration benzene removal. (2) The defect engineering strategy for oxygen vacancy creation. (3) The structure-activity relationship governing photocatalytic performance. The as-developed SiO_2_/TiO_2_-based photocatalytic material was evaluated for both low-concentration benzene degradation and antimicrobial inactivation, demonstrating dual functionality for organic pollutant removal and microbial disinfection. Under UV irradiation, benzene degrades 89% by T@S NPs within 30 min, a three-fold increase over that of pristine TiO_2_. A 99.5% benzene degradation was achieved within 60 min and demonstrated a stable photocatalytic activity over five cycles. Notably, coating TiO_2_ with amorphous SiO_2_ rendered a nearly neutral characteristic of T@S NPs. They displayed high stability, notable adhesion, and robust photocatalytic antibacterial property, highlighting their promising potential for practical photocatalytic applications.

## 2. Experimental Section

### 2.1. Materials

Titanyl sulfate (TiOSO_4_·xH_2_SO_4_·xH_2_O, >93%), sodium hydroxide (NaOH > 99.0%), and hydrogen peroxide (H_2_O_2_, >30%) were purchased from Sinopharm Chemical Reagent Co., Ltd., Shanghai, Chian. Tetraethyl orthosilicate (Si(OC_2_H_5_)_4_, 99%) was used as SiO_2_ sources and obtained from Shanghai Macklin Biochemical Co., Ltd., Shanghai, Chian. All the chemicals were used without further purification.

### 2.2. Synthesis of Mesoporous SiO_2_-Coated TiO_2_ Nanoparticles

All powder samples used in the following tests were vacuum dried at 60 °C. Synthesis of SiO_2_-coated TiO_2_ nanoparticles (S@T NPs) was carried out via a sol-hydrothermal process following our previous work. An aqueous solution of titanium sulfate (0.2 mol/L) was prepared as the titanium source. A 0.01 mol/L sodium hydroxide solution was then added dropwise under continuous stirring, inducing the formation of a white precipitate. The pH of the solution was carefully adjusted to 5–6. The precipitate was subsequently purified through repeated washing and centrifugation (4–6 cycles) to eliminate impurities. The purified product was redispersed in deionized water and reacted with hydrogen peroxide under magnetic stirring at 40 °C for 4 h, yielding a peroxo titanic acid precursor sol. To prepare the TiO_2_@SiO_2_ composite, tetraethyl orthosilicate (TEOS) was introduced into the sol at molar ratios of 10%, 15%, and 20%, corresponding to samples labeled as 1-T@S, 2-T@S, and 3-T@S, respectively. The mixtures were subjected to hydrothermal treatment in a sealed reactor at 120 °C for 6 h, producing colloidal T@S with a near-neutral PH. Tetraethyl orthosilicate was then added to the above sol at a mole ratio of Si to Ti of 10%, 15% and 20%, respectively, and the resulting samples were denoted1-T@S, 2-T@S, and 3-T@S, followed by heating at 120 °C for 6 h in a hydrothermal reactor. For comparison, pure TiO_2_ sol was also synthesized by the same method and designated as TiO_2_.

### 2.3. Characterization

*Materials structure and components.* Crystal phases of samples were determined by X-ray diffraction (XRD, Ultima IV, Rigaku Corporation, Tokyo, Japan) with Cu target. Fourier transform infrared (FTIR) spectra of all samples were measured on a instrument (Nicolet iS20, Thermo Fisher Scientific, Waltham, MA, USA). For the preparation of TEM samples, 20 μL of the samples was dropped on a copper grid for characterization by transmission electron microscopy (TEM, JEM-F200, JEOL Limited, Tokyo, Japan). Brunauer-Emmett-Teller specific surface area was determined using a surface area analyzer (BET, Nova 2000e, Quantachrome Instrument, Boynton Beach, FL, USA) with nitrogen adsorption. X-ray photoelectron spectroscopy (XPS, K-Alpha, Thermo Fisher Scientific, Waltham, MA, USA) was performed to investigate the chemical state of elements. UV-Vis absorption spectra of samples were measured using a UV-Vis spectrophotometer (UV-Vis DRS, U4150, Hitachi Limited, Tokyo, Japan). Oxygen vacancy, superoxide anions (·O_2_^−^), and hydroxyl radicals (·OH) were investigated using electron paramagnetic resonance (EPR, A300, Bruker Corporation, Karlsruhe, Germany) spectroscopy. Photoluminescence spectra of samples were measured using a fluorescence spectrophotometer (PL, FLS 1000, Edinburgh Instruments, Livingston Village, Scotland).

*Photodegradation performance.* A home-built gas phase degradation system shown in Figure S1 is divided into gas distribution, reaction, and analysis systems. The continuous analysis was performed using Shimadzu’s gas chromatograph-mass spectrometer (GC-MS 2010, Shimadzu, Tokyo, Japan), which was sampled every half hour. The samples were placed in the gas phase photocatalytic reaction chamber. The gas in the chamber was replaced with oxygen, and a 254 nm xenon lamp was used to pretreat for 30 min to remove pollutants adsorbed on the surface of the samples. The gas in the cabin was replaced with oxygen many times, and the gas was further distributed to ensure the concentration range of benzene is approximately 10 ppm. The carrier gas is oxygen, and the chamber energy pressure is atmospheric pressure. First, dark adsorption was carried out for 1 h to allow the adsorption and desorption process to reach equilibrium. The photocatalytic reaction process was carried out for 4 h, with a xenon lamp as the light source (wavelength ≥ 380 nm and optical density of 50 mW/cm^2^).

*Adhesion performance.* The adhesion performance of the material was characterized using a scrub resistance experiment. Material was sprayed, and a smooth glass sheet was loaded with 20 mg per piece. The glass sheet was placed on the BEVS 2805 type scrub resistance tester, and the scrubbing experiment was carried out with a natural brush loaded with a 450 g weight. After scrubbing 200, 500, and 1000 times, the morphology of the coating was imaged using a UM203i microscope. The remaining material amount was compared, and the adhesion performance was evaluated.

*Antibacterial performance.* Bacterium *Escherichia coli* 25922 (*E. coli*) was used to test the antibacterial performance. The detailed experimental method can be found in the Supporting Information.

## 3. Results and Discussion

### 3.1. Microstructure and Morphology of the TiO_2_@SiO_2_ Nanoparticles

**Figure 1:**
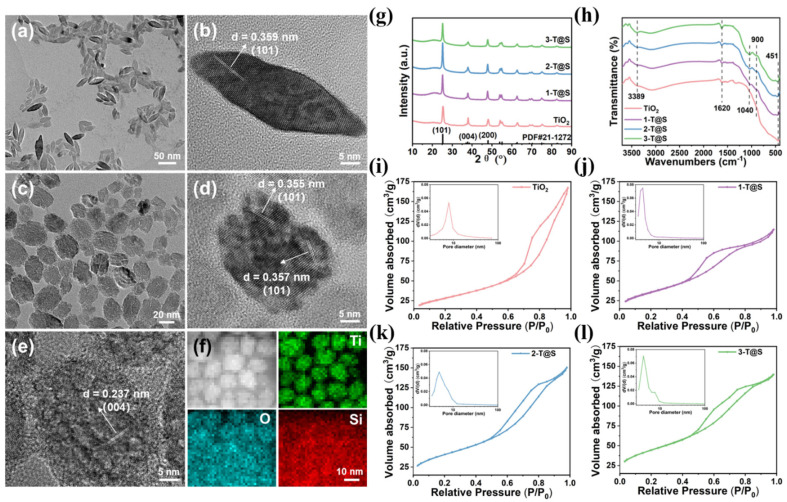
TEM images for: (**a**,**b**) TiO_2_ NPs; (**c**–**e**) 2-T@S NPs; (**f**) Element mappings of 2-T@S NPs (**g**) XRD patterns of TiO_2_ and T@S NPs; (**h**) FTIR spectra of TiO_2_ and T@S NPs (**i**–**l**) N_2_ adsorption-desorption isotherm and the pore size distribution curve of all samples.

Figure 1a shows the fusiform morphology of pristine TiO_2_ NPs. The lattice spacing was found to be 0.36 nm (Figure 1b), corresponding to the (101) crystal plane of anatase phase TiO_2_. The introduction of SiO_2_ led the fusiform-shaped TiO_2_ to transition into spherical shape, forming SiO_2_-coated TiO_2_ nanoparticles (denoted 2-T@S NPs, see Section 2) (Figure 1c–e). The clarity of the lattices in T@S composite NPs is reduced, suggesting the formation of amorphous SiO_2_ over TiO_2_ NP with the exposed crystal plane of the latter remaining to be the (101) crystal face.

The element surface sweep (Figure 1f) reveals that the Ti element exists within nanoscopic spheres, while the Si element is relatively dispersed, signifying that T@S NPs have an amorphous SiO_2_ coat on the TiO_2_ surface. The analysis of the morphology shows that the as-prepared pristine TiO_2_ and 2-T@S NPs have a major axis of 40.9 nm and 30.5 nm, respectively, and a draw ratio of 3.1 and 1.3, respectively. Clearly, the major axis shortened (Table 1).

The XRD patterns of the as-prepared samples are shown in Figure 1g. All diffraction peaks can be indexed to anatase TiO_2_ (JCPDS No. 21-1272), suggesting the crystal structure of TiO_2_ was not affected by the introduction of SiO_2_ in 2-T@S-15. As seen in Figure S2, broad peaks occur at 20–30° in the XRD patterns of pure SiO_2_ colloids at various hydrothermal temperatures instead of acute absorption peaks, suggesting that Si presents on the surface of TiO_2_ in an amorphous state, consistent with the previous TEM analysis. In addition, it is clear that the 2-T@S sample had the highest crystallinity with the (101) crystal plane diffraction peak being more intense than that of pristine TiO_2_. Thus, the addition of SiO_2_ precursor during TiO_2_ growth promoted the (101) crystal surface growth (see Section 2).

Figure 1h shows the FTIR spectra of different samples, where 3389 cm^−1^ and 1620 cm^−1^ belong to the telescopic and bending vibration peaks of surface hydroxyl groups, respectively [12]. 451 cm^−1^ corresponds to the bending vibration peak of Ti-O-Ti. It is evident that the elastic vibration peak of Ti-O-Ti increases first, and then decreases with the increase in SiO_2_ content. With SiO_2_ incorporation, the telescopic vibration of Si-O-Si appeared at 1040 cm^−1^, and the telescopic vibration of the Si-O-Ti bond at 900 cm^−1^ is clearly observed, indicating the coating of SiO_2_ and a chemical link with TiO_2_ [21].

Figure 1i–l depicts the N_2_ adsorption-desorption isotherm of the as-prepared samples, where the insets are the pore size distribution curves. The adsorption-desorption behavior of samples follows a type IV isotherm. In the low-pressure region (P/P_0_ < 0.4), there is a linear relationship between the adsorption amount and the partial pressure, which may occur in physical adsorption in a monolayer. When P/P_0_ > 0.4, the adsorption capacity increases sharply, displaying a typical behavior of materials with mesopores [13]. As P/P_0_ continues to increase, a hysteresis loop appears at high partial pressure [22]. The hysteresis loops of adsorption isotherms of all composites appeared in the medium and high pressure regions, indicating that there are mesopores and macropores within materials, while the difference is that the TiO_2_ adsorption isotherm curve was H2b type [23]. With the increase in SiO_2_ content, the T@S composite materials became H4 type, signifying the emergence of micropores with the pore size distribution widened. Clearly, the T@S composites showed the coexistence of micropores and mesopores, substantiating that the coating of SiO_2_ on the TiO_2_ surface promoted the formation of more micropores.

The average pore size of TiO_2_ NPs was calculated by Barrett-Joyner-Halenda (BJH) to be 7.91 nm. The average pore size of the three T@S NPs was 4.345, 4.913, and 4.325 nm, respectively (Table 1). The specific surface area increased from 102.23 cm^2^/g (TiO_2_) to 160.52 cm^2^/g (3-T@S), verifying that the amorphous SiO_2_ coating on the TiO_2_ surface significantly increased the specific surface area of the resulting composites.

**Figure 2:**
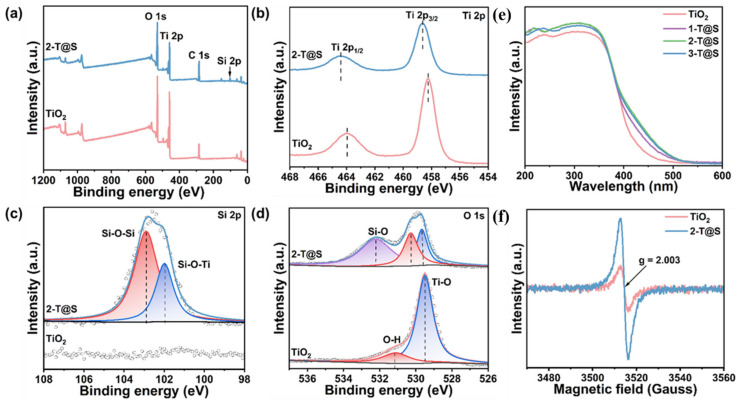
XPS spectra of (**a**) the survey; (**b**) Ti 2p; (**c**) Si 2p; (**d**) O 1s for TiO_2_ and 2-T@S. (**e**) UV-Vis absorption for all samples. (**f**) EPR spectra of oxygen vacancy for TiO_2_ and 2-T@S.

The binding energies at 463.75 eV and 458.02 eV in the XPS spectra of Ti 2p correspond to the Ti 2p_1/2_ and Ti 2p_3/2_ orbitals, respectively (Figure 2b), confirming the formation of anatase TiO_2_ and the presence of Ti in the Ti^4+^ oxidation state [14,15]. For 2-T@S NPs, the Ti 2p orbital has a significant blue shift, and the binding energies at the Ti 2p_1/2_ and Ti 2p_3/2_ orbitals increase. This is because the greater electronegativity of Si than that of Ti reduces the electron density around Ti atoms [24]. In Figure 2c, the Si 2p peak of 2-T@S NPs can be deconvoluted into two peaks. They are the Si 2p orbital of Si-O-Si and Si-O-Ti [25], respectively. The main peak of Si 2p is 102.90 eV, reflecting the Si characteristic in the SiO_2_ tetrahedron [26], and SiO_2_ tends to grow uniformly on the surface of TiO_2_. The O 1s spectrum can be deconvoluted into three peaks (Figure 2d). The first peak is attributed to oxygen in SiO_2_, the second peak is the hydroxyl group on the surface, and the third peak is from lattice oxygen of Ti [27]. Clearly, the proportion of O 1s peaks generated by hydroxyl groups becomes larger, indicating that the introduction of Si enhances the surface electrification of TiO_2_-based photocatalytic materials, which is conducive to the adsorption of organic pollutants for catalytic oxidative degradation reactions. The overall peak shift of O 1s indicates that there may be oxygen vacancies on the surface due to lattice distortion [28]. In addition, according to the peak fitting results, the hydroxyl oxygen content in pristine TiO_2_ is 16.98%, while it reaches 26.72% in 2-T@Scomposites. This result suggests a potentially improved photocatalytic performance of T@SNPs over that of TiO_2_; the increase in surface hydroxyl groups can provide more active sites and promote the generation of free radicals, thus improving the photocatalytic efficiency [26].

UV-vis absorption spectroscopy was performed on all the samples (Figure 2e). An obvious absorption peak in the wavelength range of 200–380 nm was observed for all the samples, corresponding to the absorption of anatase phase TiO_2_. Compared to pristine TiO_2_, the absorption ability of T@S composites at different Si/Ti ratios in the UV region was nearly the same. The light absorption in the visible region was slightly improved. This is due to the introduction of oxygen vacancies in the TiO_2_ matrix, resulting in new electronic states in the band gap [29]. Overall, the introduction of SiO_2_ has little effect on the absorbance and band gap of TiO_2_-based photocatalytic materials [30].

### 3.2. Photocatalytic Performance of T@S NP Composites

#### 3.2.1. Benzene Degradation

**Figure 3:**
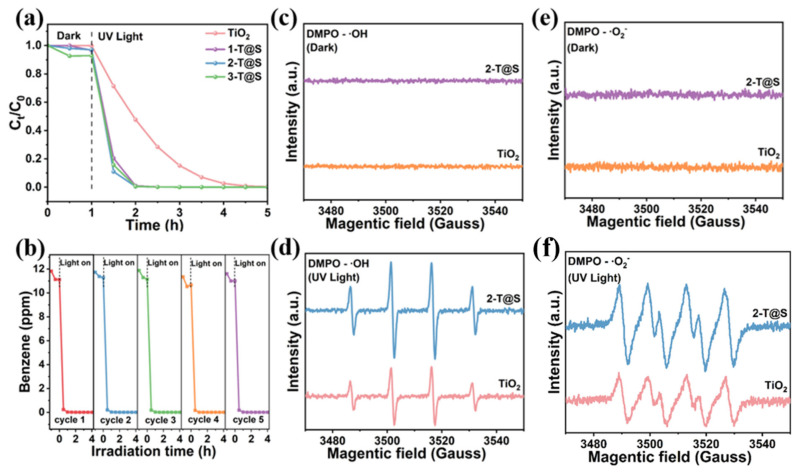
(**a**) Photocatalytic degradation of benzene by pristine TiO_2_ NPs and three T@S NPs. (**b**) Cycle stability testing of benzene degradation by 2-T@S. (**c**–**f**) DMPO-•OH spectra of pristine TiO_2_ and 2-T@S measured with electron paramagnetic resonance (EPR). DMPO-•OH: (**c**) in dark, and (**d**) with UV light. DMPO-•O2^−^: (**e**) in dark, and (**f**) with UV light.

The photocatalytic performance of the as-prepared T@S NPs was studied by UV-vis degradation of benzene (Figure 3a). The pristine TiO_2_ NPs almost do not adsorb benzene in the dark in the first hour. For T@S NPs with the increasing SiO_2_ content, the benzene concentration declined, demonstrating a progressively enhanced adsorption capacity. Thus, T@S NPs resolve the issues of pristine TiO_2_, that is, limited adsorption of benzene and light of the latter. This is due to the formation of mesoporous SiO_2_ (Figure 1j–l), leading to increased specific surface area, as well as the adsorption of benzene molecules by acidic sites (Figure S3) as a result of the formation of Ti-O-Si bonds in T@S NP [31,32,33].

After 4-h UV-visible irradiation, pristine TiO_2_ and three T@S composites reached a 99.9% degradation of benzene. Notably, within the first 1 h of illumination, the degradation ability of T@S composites for benzene is much higher than that of pristine TiO_2_. The degradation rate of benzene is 28.74%, 79.62%, 88.96%, and 84.32% for pristine TiO_2_, 1-T@S, 2-T@S, and 3-T@S, respectively, when exposed to 30 min. Clearly, the degradation by 2-T@S is the fastest, representing 3.1-fold over pristine TiO_2_. After 1 h of UV-vis exposure, 2-T@S composites achieved a 99.5% benzene degradation.

Figure 3b depicts the excellent cycling stability of 2-T@S. The benzene degradation efficiency is retained over a five-cycle process, achieving a 99% benzene removal with no tail warping after 1 h of UV-vis irradiation.

#### 3.2.2. Mechanisms of Enhanced Photocatalytic Activity

In the photocatalytic degradation of benzene and other gaseous organic pollutants, the amount of hydroxyl radical and superoxide radical produced is closely related to the photocatalytic degradation rate.

In the electron paramagnetic resonance (EPR) study (Figure 3c–f), 5,5-dimethyl-1-pyrroline N-oxide (DPMO) was used as a trapping agent to capture hydroxyl and superoxide radicals in different organic carriers. Under the dark condition, no reactive oxygen species were generated in the samples. After UV-visible (λ > 380 nm) irradiation for 5 min, hydroxyl and superoxide radicals can be detected in both TiO_2_ and 2-T@S samples. The greater peak intensity of 2-T@S underscores that the surface modification with SiO_2_ promotes the generation of reactive oxygen species in T@S composites. On the one hand, the increased surface hydroxyl group and photogenic electron reaction, compared with the reaction with water molecules, require a lower reaction energy and can more readily produce hydroxyl radicals. On the other hand, during the high-temperature treatment process, SiO_2_ coating will restrict the contact between TiO_2_ and oxygen, creating a local hypoxic environment. At the same time, the chemical reaction between the hydroxyl groups (-OH) on the SiO_2_ surface and the lattice oxygen of TiO_2_ jointly causes TiO_2_ to lose its lattice oxygen, thereby forming oxygen vacancies. The presence of oxygen vacancies is favorable for the separation of photogenerated electron-holes and further improves the photocatalytic performance [34]. It is evident that the oxygen vacancies in 2-T@S measured with EPR (Figure 2f) are higher than those of pristine TiO_2_.

**Figure 4:**
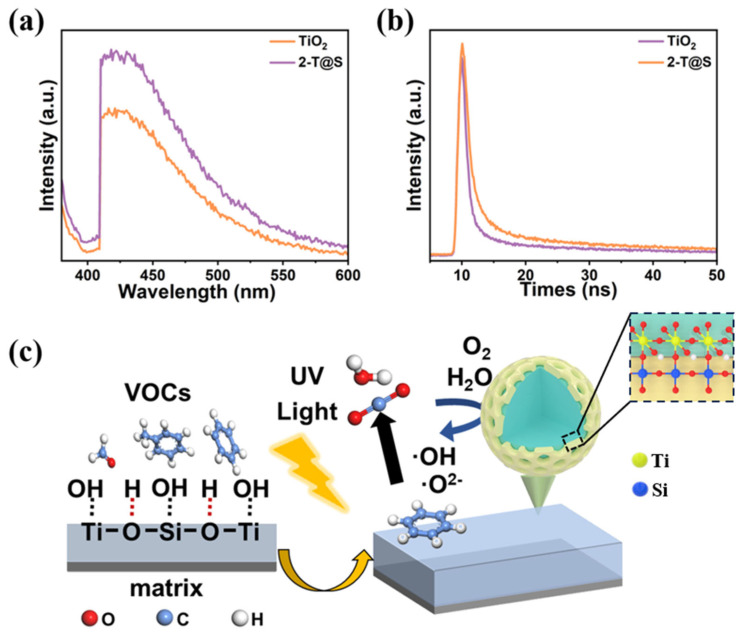
(**a**) Steady-state fluorescence spectra of pristine TiO_2_ and 2-T@S. (**b**) Carrier lifetime spectra of pristine TiO_2_ and 2-T@S. (**c**) Schematic illustration of the mechanism of enhanced benzene degradation enabled by T@S NPs.

Figure 4a compares the steady-state fluorescence spectra of the as-prepared samples with the excitation wavelength of 380 nm and the emission wavelength of 425 nm. The similar fluorescence spectra of TiO_2_ and 2-T@S indicate that the introduction of SiO_2_ did not result in a new fluorescence peak. The increase in fluorescence intensity of 2-T@S in the range of 425 nm to 500 nm can be attributed to the generation of free excitons with edges, and the 2-T@S sample has more oxygen vacancies. The exciton energy levels close to the conduction band minimal can be formed, combined with photoelectrons to form excitons, thereby enhancing the PL signal. In the photocatalytic process, surface oxygen vacancies and defects are conducive to capturing photogenerated electrons. The enhanced fluorescence intensity signifies the improved photocatalysis [35].

The carrier lifetime was measured with time-resolved fluorescence spectroscopy (Figure 4b) and fitted by the second-order exponential equation of the curve. Among them, the short-lived τ_1_ is related to the fluorescence of the free exciton, while the long-lived τ_2_ is attributed to the trap exciton of the surface defect state. Comparing the relationship between A_1_ and A_2_, the lifetime is mainly owing to the intrinsic fluorescence (τ_1_), while the contribution of surface defects is small (τ_2_). The overall carrier lifetimes of TiO_2_ and 2-T@S are 0.83 ns and 0.89 ns, respectively (Table 2). The SiO_2_ coating slows down the mobility of carriers, facilitates the separation of electron-hole pairs, and ultimately increases the carrier lifetime [36].

Taken together, enhanced photocatalytic degradation of benzene can be rationalized as follows (Figure 4c). First, the crafting of mesoporous SiO_2_ on the TiO_2_ surface increases the specific surface area of T@S composites, leading to the improved adsorption of benzene. Second, the amount of hydroxyl groups on the surface of T@S composites increases, which is conducive to the production of reactive oxygen radicals. Third, the presence of ample oxygen vacancies in T@S composites introduces defect energy levels at the conduction band minimal of TiO_2_ and improves the light response ability.

### 3.3. Practical Application Potentials

In addition to effective benzene removal by T@S composites due to their excellent photocatalytic activity, their stability and adhesion properties were examined to further explore their practical application potentials.

#### 3.3.1. Stability of T@S NP Composites

**Figure 5:**
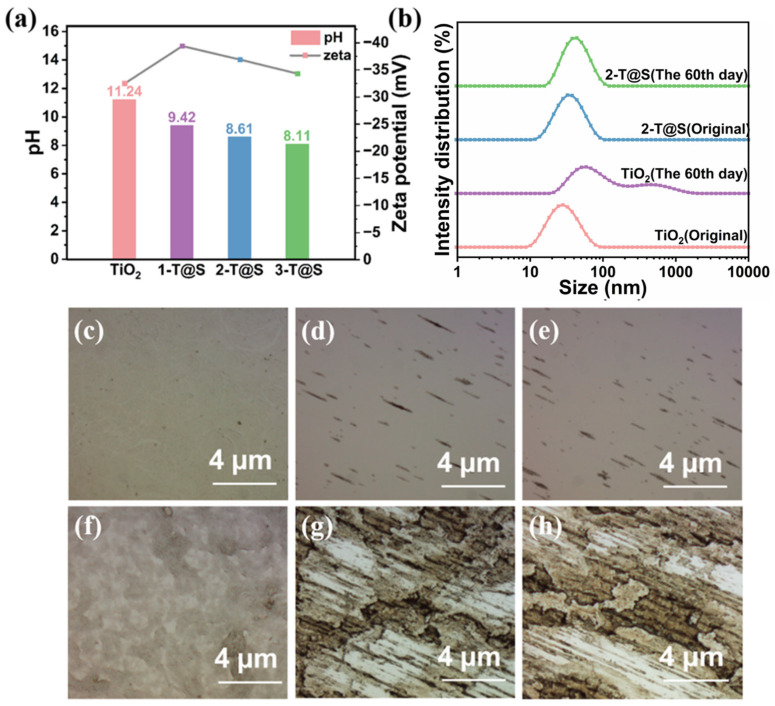
(**a**) pH value and Zeta potential of pristine TiO_2_ and three T@S NPs. (**b**) NP size distribution of pristine TiO_2_ and T@S NPs before and after 60-day storage. (**c**–**h**) Micrographs of (**c**–**e**) pristine TiO_2_ and (**f**–**h**) 2-T@S after different scrubbing times: (**c**,**f**) before scrubbing; (**d**,**g**) scrubbed for 200 times; and (**e**,**h**) scrubbed for 1000 times.

Figure 5a compares the zeta potential and pH value of pristine TiO_2_ and T@S NPs. Clearly, pristine TiO_2_ NPs are alkaline with a pH value of 11 and a zeta potential greater than −30 mV. This is because there may be non-crystallized peroxytitanates on the anatase TiO_2_ surface. Thus, in the colloidal state, there are -OH groups and -OOH groups on the TiO_2_ surface. The surface is negatively charged, and a surface electrical double layer can be formed in an aqueous solution. As such, there exists a large repulsive force between NPs, leading to good stability.

Compared with pristine TiO_2_ NPs, the pH value of T@S NPs is decreased due to the hydrolysis reaction between the surface Si-OH in SiO_2_ and water molecules, releasing H^+^, thus reducing the solution alkalinity. The pH value of T@S NP solution and the electrostatic repulsion caused by the presence of abundant hydroxyl groups on the surface of TiO_2_ and SiO_2_, make the zeta potential of T@S NPs still larger than −30 mV. Taken together, T@S NPs display excellent stability while being close to neutral (pH = 8.11–9.42; Figure 5a).

To verify the long-term stability of NPs, the sample was placed in an indoor environment for 60 days. Neither pristine TiO_2_ nor T@S NPs showed precipitations (Figure S4). The zeta potentials of pristine TiO_2_ gel and T@S gel are −31.4mV and −32.8 mV, respectively. Figure 5b shows the NP size after 60-day storage. For pristine TiO_2_ gel with an original average size of 42.2 nm, two peaks at 38.15nm (primary peak) and 215.7 nm (secondary peak) were seen after 60 days. The secondary peak is relatively small, signifying that TiO_2_ NPs slowly agglomerate during storage. In contrast, the secondary particle size of T@S is 37.35nm, which accords with normal distribution. With the increasing storage time, the stability of T@S NPs is enhanced. The secondary particle size measured by the dynamic light scattering laser particle size analyzer (DLS) was within the range of 20–40 nm, and it increased with the increase of the TEOS introduction amount (Table 3). Moreover, no large peak was observed, which proved that the long-chain peroxy titanium acid completely crystallized after the hydrothermal treatment.

#### 3.3.2. Adhesion Characteristic of T@S NP Composites

The adhesion of the as-prepared samples on glass was evaluated by brush scrubbing using an optical microscope in the transmission mode, where the bright region suggests that the material was removed during the scrubbing process. The specific coating preparation can be found in the Supplementary Information.

Figure 5c,f show the optical micrograph of the original TiO_2_ and T@S samples, respectively, indicating a uniform surface. Pristine TiO_2_ on the glass substrate had nearly completely disappeared after 200 times of scrubbing. In contrast, 2-T@S composites exhibit a superior scrubbing resistance. After 1000 times of scrubbing, 2-T@S composites still remain on the glass surface. It is notable that 2-T@S composites may form ample chemical bonds (i.e., Ti-O-Si) with the glass substrate (possessing Si-O-Si bonds), thereby enhancing the adhesion performance of photocatalytic T@S composites.

### 3.4. Antibacterial Characteristic of T@S NP Composites

**Figure 6:**
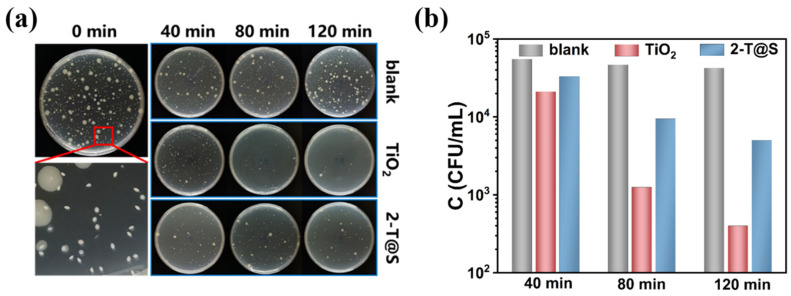
(**a**) Photos of *E. coli* colony inactivated by pristine TiO_2_ and 2-T@S NPs under UV irradiation. (**b**) Map of *E. coli* colony inactivation by pristine TiO_2_ and T@S NPs under UV irradiation.

Figure 6a shows the effect of colony activity of *E. coli* treated by pristine TiO_2_ and 2-T@S samples at different times in a constant temperature incubator under UV irradiation. At time zero, most *E. coli* was found to form rod-shaped, relatively small colonies, where rod-shaped *E. coli* grew inside the agar. In addition, there are some prominent round-shaped colonies on the surface of agar that are in contact with more water and are easier to grow into circles. The colony data of the control sample remained within 10^5^ orders of magnitude within 120 min, suggesting that the colony had good activity, and UV irradiation had a negligible effect on the colony growth. On aluminum plates coated with pristine TiO_2_ and T@S photocatalytic NPs, a decrease in the number of colonies was observed.

As shown in Figure 6b and Table 4, the antibacterial performance of pristine TiO_2_ NPs is superior to that of T@S NP composites. TiO_2_ NPs achieve a sterilization effect of 99.4% after 120 min, while the sterilization ability of T@S composites is slightly reduced (92.6%). The weak antibacterial effect of T@S may be due to the change of surface state. The pH value of pristine TiO_2_ NP solution is higher than that of T@S NP solution(shown in Figure 5a), which has a higher equipotential and a stronger surface alkalinity, thus being unfavorable for the survival of *E. coli*. In addition, TiO_2_ NP solution has a destructive effect on *E. coli*, changing the osmotic pressure of the cell membrane and thereby dissolving intracellular substances. However, after coating or compositing on the surface of SiO_2_, the osmotic dissolution effect of T@S NP is weakened, so the antibacterial efficiency of T@S NPs decreases. In conclusion, the results of the antibacterial property test indicate that T@S NPs have the effect of photocatalytic inactivation of microorganisms, yet the antibacterial rate is weakened due to the surface coating or compounding of SiO_2_.

## 4. Conclusions

In summary, we developed a viable route to photocatalytic benzene removal enabled by enhanced adsorption using rationally designed mesoporous SiO_2_-coated TiO_2_ NPs (T@S). The T@S NPs in their colloidal form can be readily dispersed, circumventing the agglomeration issue as widely encountered in powder form. The morphology of T@S NPs with (101) crystal lattice exposed changes from a spindle shape in pristine TiO_2_ NPs to a spherical shape. As revealed by TEM, XPS, FTIR, and EPR studies, the SiO_2_ coating over TiO_2_ NP is amorphous, and SiO_2_ and TiO_2_ form a Ti-O-Si bond. The T@S NPs possess ample micro- and meso-pores, oxygen vacancies, and Ti-O-Si bonds, which synergistically promote photocatalytic degradation activity. The benzene degradation cycle test unveils that benzene removal by T@S NPs reaches 99.5% within 60 min, and maintains 99.9% within 2 h after 5 cycles, suggesting their long-term degradation ability. In addition, T@S NPs display nearly neutral characteristic compared to alkaline pristine TiO_2_ NPs, and thus are noncorrosive to glass substrates. T@S NPs also demonstrate good adhesion and long-lasting antibacterial properties. Our study highlights the potential of constructing mesoporous SiO_2_-coated semiconductor nanocomposites for a wide range of practical photocatalysis applications.

## Figures and Tables

**Table 1:** Specific surface area, pore volume, and pore diameter of all samples.

	Surface Area (m^2^/g)	Pore Volume (cm^3^/g)	Pore Diameter (nm)
TiO_2_	102.23	0.26	7.91
1-T@S	128.32	0.16	4.35
2-T@S	146.74	0.22	4.91
3-T@S	160.52	0.20	4.33

**Table 2:** Carrier lifetime fitting of TiO_2_ and 2-T@S.

Sample	τ_1_ (ns)	τ_2_ (ns)	A_1_	A_2_	τ (ns)
TiO_2_	0.847	0.0001232	98.01%	1.99%	0.83
T@S	0.9073	0.0001541	98.42%	1.58%	0.89

**Table 3:** Transparency and secondary particle size data of TiO_2_ and TiO_2_@SiO_2_ colloid measured with DLS.

	TiO_2_	1-T@S	2-T@S	3-T@S
**Transparency (%)**	77.5	74.1	68.5	66.1
**the Secondary Particle Size (nm)**	25.7	28.5	32.0	33.6

**Table 4:** Antibacterial performance (i.e., inactivating *E. coli*) of pristine TiO_2_ and 2-T@S.

Sample	Bacteriostatic Percentage After 40 min (%)	Bacteriostatic Percentage After 80 min (%)	Bacteriostatic Percentage After 120 min (%)
TiO_2_	68.9	98.1	99.4
2-T@S	51.1	86.0	92.6

## Data Availability

The original contributions presented in the study are included in the article, further inquiries can be directed to the corresponding authors.

## Supplementary Materials

The following supporting information can be downloaded at: https://www.mdpi.com/article/10.3390/ma18163844/s1, Figure S1: Experimental Setup for Photocatalytic Degradation of Gaseous Benzene; Figure S2: XRD pattern of SiO_2_ colloid at different hydrothermal temperatures. (Sample 40-SiO2 is the SiO_2_ colloidal drying sample after 2 h of water bath at 40 °C, and sample 120-SiO_2_ is the SiO_2_ colloidal drying sample after further water heating at 120 °C for 6 h); Figure S3: Schematic diagram of acidic sites in SiO_2_-coatedTiO_2_ NPs; Figure S4: Digital image of TiO_2_ and 2-T@S sample stored after 60 days.
