# Supplementary material for: Robust Mesoporous SiO2-Coated TiO2 Colloidal Nanocrystal with Enhanced Adsorption, Stability, and Adhesion for Photocatalytic Antibacterial and Benzene Removal
Source: Materials (Basel). 2025 Aug 15;18(16):3844. doi: 10.3390/ma18163844 (PMC12387839; doi:10.3390/ma18163844)
Supplement: Supplementary file 1 [file materials-18-03844-s001.zip › materials-3793174-supplementary.pdf]

materials

Supporting Information for Robust Mesoporous SiO_2-coated TiO_2 Colloidal Nanocrystal with Enhanced Adsorption, Stability, and Adhesion for Photo-catalytic Antibacterial and Benzene Removal

Nan Xiao [1,2], Aijia Zhang [1,2], Kunjie Yuan [1,2]* and Wenbin Cao [1,3]*

[1] School of Materials Science and Engineering, University of Science and Technology Beijing, Beijing 100083, P. R. China; 13982936009@163.com (N.X), 19134020402@163.com (A.Z.)
[2] School of Energy and Environmental Engineering, Hebei University of Technology, Tianjin 300401, P. R. China.
[3] Tianjin College, University of Science and Technology Beijing, Tianjin 301830, P. R. China.
* Correspondence: kyuan@hebut.edu.cn (K.Y.); wbcao@ustb.edu.cn (W.C.)

Photocatalytic degradation performance of benzene

The performance test of the photodegradation of benzene mainly aims to analyze the photodegradation performance of photocatalytic materials for benzene. The benzene degradation experiment in this paper was conducted in a static reaction experimental device that was self-built. The concentration changes of benzene were monitored online using the GC-MS2010 gas chromatography–mass spectrometry instrument produced by the Shimadzu Corporation of Japan. The schematic diagram of the experimental device is shown in Figure S1.

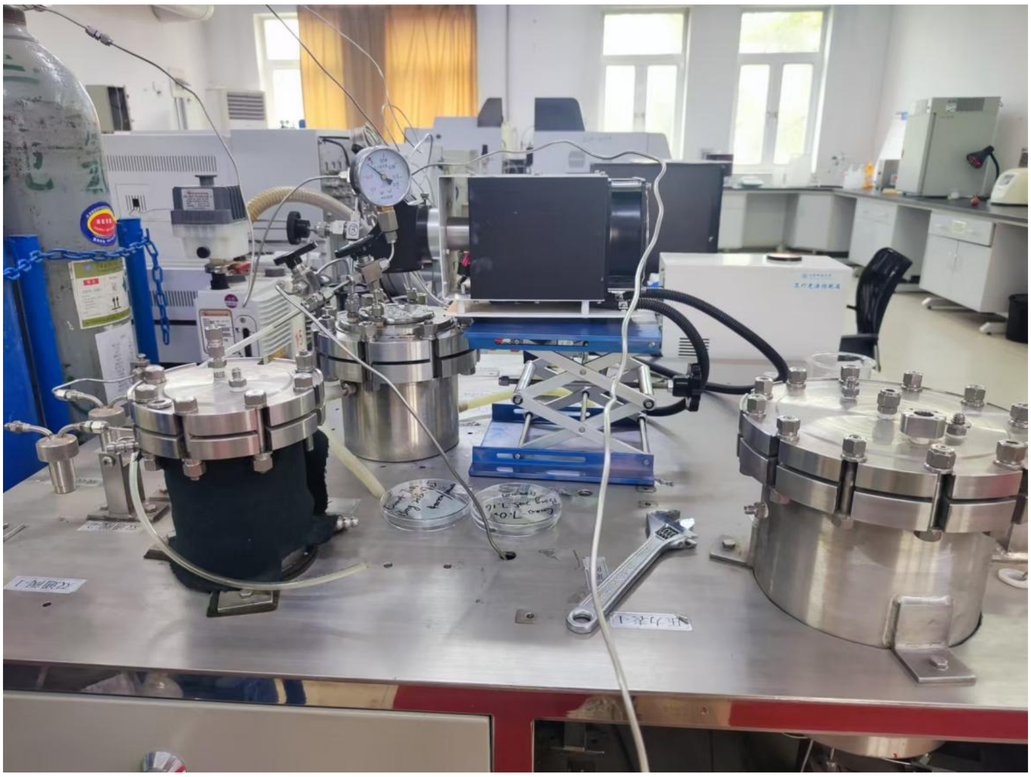

Figure S1. Experimental setup for photocatalytic degradation of gaseous benzene.

The prepared sol sample was sprayed onto a clean glass plate with a size of 50 mm x 50 mm using a high-pressure spray gun. After spraying, the sample was dried at 80 ° C using an electric heating platform. The spraying and drying process was repeated, and the weight was measured. Finally, the mass of the photocatalyst coated on the glass sheet was controlled to be approximately 12.5 mg (0.5 mg/cm^2), and the sample for testing was prepared. The quality of the photocatalyst powder and the coverage area on the glass substrate will also affect the photocatalytic efficiency to a certain extent. A low loading amount will result in insufficient active sites for the contact of benzene molecules, thereby reducing the degradation efficiency. If the glass surface is sprayed unevenly (such as the presence of uncovered areas), it will directly reduce the effective catalytic area. Therefore, the quality of the powder (loading amount, dispersion, oxygen vacancy concentration) and the coverage area of the substrate (uniformity, geometric matching) will, to some ex-tent, influence photocatalytic efficiency. Coordinated optimization is required to achieve the best catalytic performance.

The test sample was loaded into a 1.5 L circular photocatalytic reactor, which was then sealed and checked for airtightness. Prior to the reaction, the system was pretreated with a 254 nm xenon lamp for 30 min to eliminate potential organic contaminants adsorbed on both the reactor and sample surfaces. The gas phase inside the chamber was repeatedly purged and replaced with oxygen to regulate the gas composition, maintaining a benzene concentration of 10 ppm. Oxygen served as the carrier gas, and the chamber pressure was equilibrated to atmospheric pressure. To establish adsorption–desorption equilibrium, the reactor was kept in the dark for 60 min. Subsequently, the photocatalytic reaction was initiated under irradiation from a xenon lamp ($\lambda \geq 380$ nm) with an incident light intensity of 50 mW/cm^2 for a duration of 4 h. Throughout the reaction, benzene concentration was monitored in real time at 30 min intervals using online GC-MS.

The photocatalytic reaction was conducted with the sample positioned 20 cm from the light source. A xenon lamp ($\lambda \geq 380$ nm) served as the irradiation source, equipped with an infrared absorption filter to eliminate thermal effects. To maintain a stable reaction temperature, the system was actively cooled by circulating chilled water throughout the experiment.

Antibacterial performance characterization

The experiment was primarily conducted on a sterile operating table equipped with ultraviolet sterilization capability. The bacteria utilized in experiment was Escherichia coli 25922 (E. coli). The culturing process took place within a light incubator set to 37 °C. A TLD lamp emitting light frequencies ranging from 380 to 800 nm with an intensity of 1.4 mW/cm^2 was employed as the light source for both culturing and experimental procedures. To establish a control group in a dark environment, the AGAR medium can be enveloped in tin foil. The materials required for the experiment are either subjected to vertical high-pressure steam sterilization at 120 °C for one hour or sterilized through UV irradiation and alcohol elimination.

(1) Preparation of antibacterial sample plate

The aluminum plate measuring 5×5 cm^2 was initially cleaned and subjected to ultrasonic treatment using deionized water and anhydrous ethanol for 30 min each. Subsequently, the gel of the test sample was sprayed onto the aluminum plate with a spray gun, applying a load of 0.5 mg/cm^2 on a heating table set to 80 °C.

AGAR medium preparation: Dissolve 2.5g AGAR in 100 mL water to boil while stirring thoroughly. Autoclave the solution and set it aside. Add approximately 3–5 mm of AGAR into each medium.

Eluent preparation: To prepare the eluent solution, add 0.25 mL Tween-80 to 500 mL deionized water. Adjust the pH value by adding potassium dihydrogen phosphate and sodium chloride until reaching a final pH range between 6.0 and 6.5.

(2) Strain culture and transfer

The initial strain of this experiment was the second-generation standard strain of E. coli 25922 purchased from Beijing Biologics. A single colony was scraped from the third line of the culture dish prepared using the plate scribing method with an inoculation ring and continued solid-phase culture. The colony was then transferred to a new AGAR medium using the four-zone line method and cultured in a light incubator. It can be transferred once every 20–24 h, which means it is considered as the next generation of bacteria.

(3) Preparation of antibacterial bacterial suspension

Active bacteria from generations 2 to 7 were scraped using an inoculation ring, and a half-ring amount was added to the inner wall of a test tube without touching its deionized water content. The colony concentration was measured by turbidimetry following national standards where OD600 = 0.5–0.65 corresponds to a colony concentration range between (1–5) ×108 cells/mL. To measure absorbance at 600 nm, adjust the fractional photometer accordingly and record it as a bacterial solution absorbance within a range of 0.5–0.65, representing a bacterial concentration of approximately 108 cells/mL.

Take the 1 mL bacterial solution and dilute it further three times with deionized water (9 mL each time), resulting in recorded bacterial concentrations of approximately 107 cells/mL, 106 cells/mL, and 105 cells/mL, respectively.

(4) Antibacterial experiment process

The time zero blank control sample was prepared. The bacterial suspension with a concentration of 105 bacteria/mL was used as the initial colony concentration without dilution, i.e., a 100-fold dilution. A volume of 200 μL of the bacterial solution was taken and added dropwise to 10 mL of eluent, labeled as a 10^{-1} dilution with a bacterial concentration denoted as Ct-1. The bacterial solution was further diluted to obtain the 10^{-2} dilution (bacterial concentration recorded as Ct-2) and the 10^{-3} dilution (bacterial concentration recorded as Ct-3). These diluted solutions were cultured for 24 h, and then drops of each dilution gradient (1 mL each) were added onto Petri dishes containing agar at 45 °C. After thorough shaking, three parallel samples were prepared for each concentration gradient.

Next, droplets of the bacterial suspension (200 μL) were added to an aluminum plate sprayed with antibacterial samples. The plate was covered with a sealing film measuring 4×4 cm^2 and incubated under light conditions for different time gradients such as 30 min or 1 h. Light and dark control groups were also set up.

After completion of the antibacterial treatment, eluent (10 mL) was added to fully elute any remaining bacteria from the plate surface. The resulting bacterial solution was further diluted to obtain both the 10^{-2} and 10^{-3} dilutions, respectively. Drops (1 mL each) from these two dilutions were added onto Petri dishes containing agar at 45 °C, followed by thorough shaking. Three parallel samples were prepared for each concentration gradient, which were then cultured in an incubator for another 24 hr before counting the number of bacteria.

(5) Colony counting method

Colony counting method on a single medium: Divide the medium into eight regions, count the number of two regions diagonally, and expand by four times to the total number of colonies.

Method 1: The plate colony counting method is used to count the three gradient colony numbers of dilution, respectively. The relationship between the three is a multiple of ten; otherwise, the experiment is regarded as human error. So, at time t, the colony concentration is

$$C_t = \frac{C_{t-1} \times 10 + C_{t-2} \times 100 + C_{t-3} \times 1000}{3}$$

where C_0 is the bacterial concentration at zero time and the C_t unit is CFU/mL. This method is relatively tedious to count and generally adopts the second method.

Method 2: Determine the dilution gradient medium with a two-digit number of colonies and obtain the average C_t of three sub-gradient repeated disks before expanding the corresponding multiple.

Thus, the antibacterial rate of the sample at time t is as follows:

$$A_t = \frac{C_0 - C_t}{C_0}$$

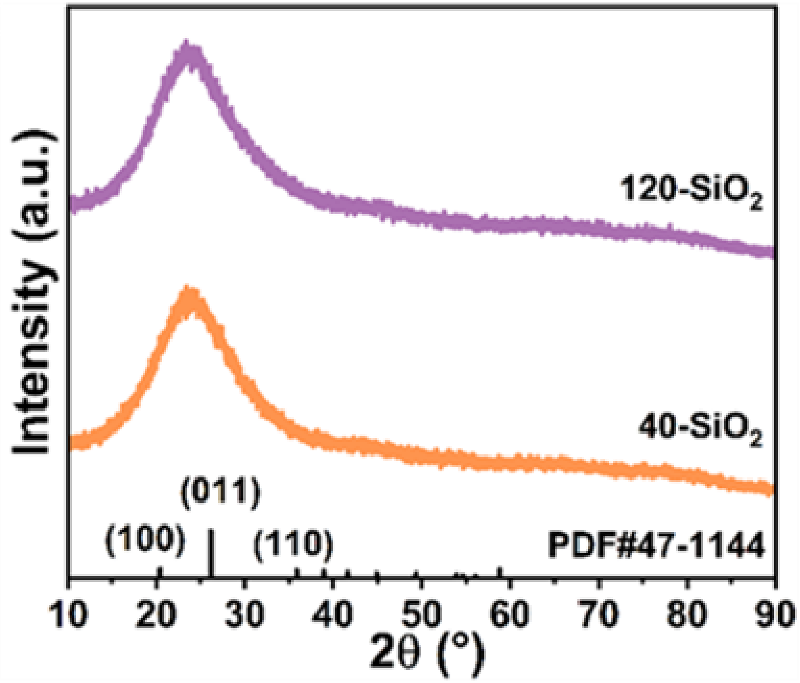

Figure S2. XRD pattern of SiO_2 colloid at different hydrothermal temperatures. (Sample 40-SiO_2 is the SiO_2 colloidal drying sample after 2 h of water bath at 40 °C and sample 120-SiO_2 is the SiO_2 colloidal drying sample after further water heating at 120 °C for 6 h.).

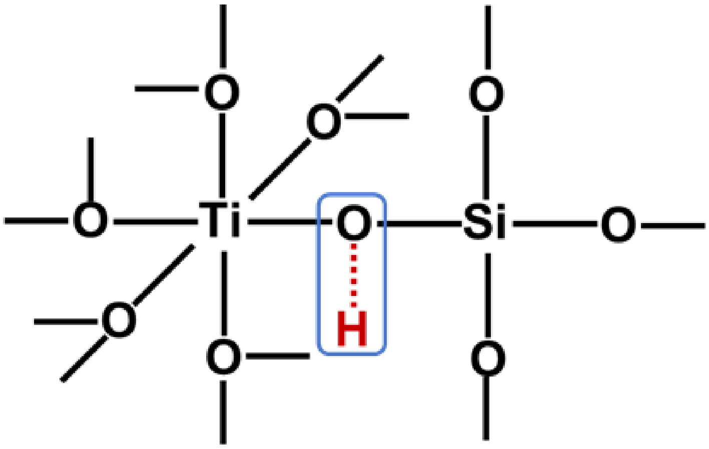

Figure S3. Schematic diagram of acidic sites in SiO_2-coatedTiO_2 NPs.

Ti-O-Si bonding connects SiO_2 and TiO_2. The site of the Ti-O-Si bond may develop into a new active center. SiO_2 is a tetrahedron, where 1 O and 2 Si create a vertical center with each of the four oxygen coordinations in Si. Ti has six coordinations, which belongs to the tetragonal structure, whereas TiO_2 is an octahedron. As such, 1 O is linked to 3 Ti. The classical intensities are

$S_{Si\text{-}O}$=4/4=1 v.u.

$S_{Ti\text{-}O}$=4/6=2/3 v.u.

Therefore, the bridge oxygen saturation between Ti-O-Si bonds is 0.33 v.u., and the Ti-O-Si site has a certain adsorption effect on organic pollutants. In addition, oxidation intermediates may be generated by TiO_2 connected to Ti-O-Si bonds. Subinclusion and bridged oxygen in Ti-O-Si can adsorb protons to form a brix hydroxyl group, which is the acidic site shown in Figure S3, thus promoting the generation of reactive oxygen species in the photocatalytic process.

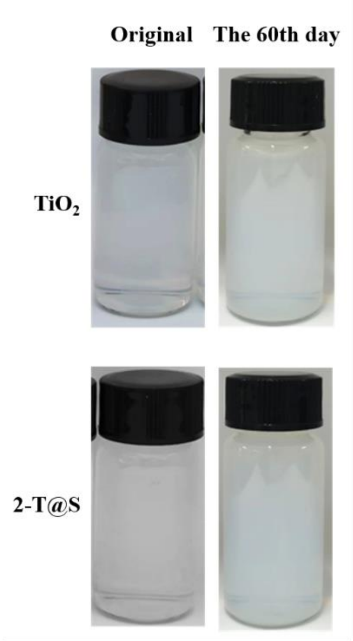

Figure S4. Digital image of TiO_2 and 2-T@S sample stored after 60 days.
